# Is surgery for recurrent lumbar disc herniation worthwhile or futile? A single center observational study with patient reported outcomes

**DOI:** 10.1016/j.bas.2022.100894

**Published:** 2022-05-11

**Authors:** Vetle Vangen Lønne, Mattis A. Madsbu, Øyvind Salvesen, Øystein Nygaard, Tore K. Solberg, Sasha Gulati

**Affiliations:** a Department of Neurosurgery, St. Olavs University Hospital, Trondheim, Norway; b Department of Neuroscience, Norwegian University of Science and Technology (NTNU), Trondheim, Norway; c Department of Public Health and General Practice, Norwegian University of Science and Technology (NTNU), Trondheim, Norway; d National Advisory Unit on Spinal Surgery, St. Olavs University Hospital, Trondheim, Norway; e The Norwegian National Registry for Spine Surgery, University Hospital of Northern Norway (UNN), Tromsø, Norway; f Institute for Clinical Medicine, UiT Arctic University of Norway, Tromsø, Norway

**Keywords:** Lumbar disc herniation, Neurosurgical procedures, Quality of life, Sciatica

## Abstract

**Objective:**

To examine outcomes and complications following microdiscectomy for recurrent lumbar disc herniation.

**Methods:**

Prospectively collected data for patients operated at the Department of Neurosurgery, St. Olavs University Hospital, Norway, were obtained from the Norwegian Registry for Spine Surgery from May 2007 through July 2016. All patients underwent lumbar microdiscectomy. The primary outcome was change in the Oswestry Disability Index (ODI) at one year. Secondary endpoints were change in quality of life measured with EuroQol 5 Dimensions (EQ-5D), back and leg pain measured with numerical rating scales (NRS), complications, and duration of surgery and hospital stays.

**Results:**

276 patients were enrolled in the study. A total of 161 patients (58.3%) completed one-year follow-up. The mean improvement in ODI at one year was 27.1 points (95% CI 23.1 to 31.0, P <0.001). The mean improvement in EQ-5D at one year of 0.47 points (95% CI 0.40–0.54, P <0.001), representing a large effect size (Cohens D ​= ​1.3). The mean improvement in back pain and leg pain NRS were 4.3 points (95% CI 2.2–3.2, P <0.001) and 3.8 points (95% CI 2.8–3.9, P <0.001), respectively. Nine patients (3.3%) experienced intraoperative complications, and 15 (5.5%) out of 160 patients reported complications within three months following hospital discharge.

**Conclusions:**

This study shows that patients operated for recurrent lumbar disc herniation in general report significant clinical improvement.

## Highlights

• Sciatica due to lumbar disc herniation (LDH) is the most common indication for spine surgery.
• There are currently limited and conflicting data on what patients can expect when undergoing repeated surgeries for recurrent LDH.
• The aim of this observational study was to investigate patient reported outcomes and complications following microdiscectomy for recurrent LDH.
• At one year we found a significant improvement in ODI-score one year after surgery. We also found significant improvements in EQ-5D-scores, leg pain NRS and back pain NRS.
• The major strengths of this study are a large number of patients, high external validity, prospective data collection, and widely applied outcome measures.

## 1 Introduction

Sciatica due to lumbar disc herniation (LDH) is the most common indication for spine surgery (Postacchini and Postacchini, 2011). Recurrent LDH with sciatica is a frequent condition with a reported incidence rate of up to 25% after the initial operation (Madsbu et al., 2018a; Lurie et al., 1976; Berjano et al., 2013; Fritzell et al., 2015). The large variations of recurrent LDH in the literature may reflect surgical technique, variability in follow-up, different definitions of recurrent LDH, and differences in access to health care (Hlubek and Mundis, 2017). Management of recurrent LDH varies, and there are no concise guidelines, only general opinions. Currently, discectomy and discectomy with fusion are the two most popular surgical options. However, there is still not enough adequate evidence in favor of either one. Even though evidence is limited, surgery is still considered to be a safe and effective alternative for patients with recurrent LDH (Shepard and Cho, 2019). There are several studies reporting incidence rates of recurrent LDH and repeat discectomy, fusion, and other treatment methods. However, few studies report patient reported clinical outcomes following repeat surgery. Those available report inconclusive result varying between comparable results to primary discectomies, no difference, and even worse outcomes (Hoogland et al., 1976; O'Sullivan et al., 1990; Papadopoulos et al., 2006; Patel et al., 2013). As a result, there are currently limited and conflicting data on what patients can expect when undergoing repeated surgical treatment for recurrent LDH.

The aim of this observational study was to investigate patient reported outcomes and complications following microdiscectomy for recurrent lumbar disc herniation.

## 2 Methods and material

### 2.1 Study population

Data were collected through the Norwegian Spine Registry (NORspine), a comprehensive nationwide registry for quality control and research (Nerland et al., 2015a). Follow-up time from the date of the last operation was one year, regardless of previous number of surgeries. We included all patients with a definitive diagnosis of symptomatic recurrent LDH who were scheduled for a single-level lumbar microdiscectomy at St. Olavs University Hospital in Trondheim, Norway between January 2007 and July 2016. All patients had undergone previous lumbar spine surgery in the same level and on the same side at least three months earlier and were all included in the NORspine registry. Patients who had coexisting degenerative spondylolisthesis and/or scoliosis were excluded, as well as patients who had previously undergone fusion surgery.

### 2.2 Data collection and registration by the NORspine registry protocol

On admission for surgery, the patients completed the self-administered baseline questionnaire, which included questions about demographics and lifestyle issues in addition to the outcome measures. During the hospital stay, using a standard registration form, the surgeon recorded data concerning diagnosis, previous lumbar spine surgery, comorbidity, *American Society of Anesthesiologists* (ASA) grade, image findings, and surgical approach and procedure. The surgeons provided data on the following possible complications and adverse events to the NORspine registry: intraoperative hemorrhage requiring blood transfusion, postoperative hematoma requiring repeated surgery, unintentional durotomy, nerve injury, cardiovascular complications, respiratory complications, anaphylactic reactions, and wrong level surgery. Patients reported the following complications if they occurred within three months after surgery: wound infection, urinary tract infection, pneumonia, pulmonary embolism, and deep venous thrombosis. A questionnaire was distributed to patients by regular mail at three months and one year after surgery, completed at home by the patients, and returned. The patients who did not respond received one reminder with a new copy of the questionnaire. The patients completed preoperative questionnaire data and postal follow-up questionnaires without any assistance from the surgeon or other staff from the treating hospital.

Information about previous or future surgery not originally registered in NORspine were collected from electronical patient journals.

## Ethical approval

The study was evaluated and approved by the regional committee for medical research in Central-Norway (2016/840), and all participants provided written informed consent.

### 2.3 Primary outcome measure

The primary outcome measure was change in disease specific functional outcome between baseline and one-year follow-up was measured with the ODI which has been translated into Norwegian and tested for psychometric properties (Fairbank et al., 1980; Grotle et al., 2003). ODI contains 10 questions on limitations of activities of daily living. Each variable is rated on a 0- to 5-point scale, summarized, and converted into a percentage score. Scores range from 0 (no disability) to 100 (bedridden). A frequently applied criteria for success is minimal disability (i.e. ODI ≤ 20 points) at one year. Others have suggested that an improvement of at least 13 points at one year could serve as a success criterion (Werner et al., 2020). A change in ODI score of less than 33% or a raw ODI score of 48 or more after surgery have been suggested as the criteria with the highest accuracy for defining failure and worsening after surgery for lumbar disc herniation (Werner et al., 2017)

### 2.4 Secondary outcome measure

Changes in health-related quality of life were measured with the Euro-Qol-5D (EQ-5D) instrument (Solberg et al., 2005). An index value for health status is generated for each patient. Scores range from −0.6 to 1, where 1 corresponds to perfect health. Effect size estimations were used to evaluate the magnitude of changes (Kazis et al., 1989).

Intensities of back and leg pain were assessed on 0 to 10 numerical rating scales (NRS), with response options ranging from 0 (no pain) to 10 (worst imaginable pain) (Jensen et al., 1992). The minimal clinically important change for NRS scales is approximately 1.5–2.0 points (Dworkin et al., 2008) (Ostelo et al., 1976). The NRS pain scales and ODI have shown good validity and are frequently used in research on back pain (Grotle et al., 2003). We also evaluated duration of procedures, length of hospital stays, repeated surgery at the index level within three months of surgery, and surgical complication rates.

### 2.5 Surgical procedures

Lumbar microdiscectomy is the favored surgical strategy for recurrent LDH at our center regardless of earlier surgery for recurrent LDH in the index level and was performed on all patients. The procedure involves preoperative fluoroscopy for detection of the target level, paramedian or median skin incision of about 3 ​cm, straight or curved opening of the paravertebral muscular fascia, and subperiosteal release of the paravertebral muscles from the spinous process and basal lamina above and occasionally below the target disc-level. Self-retaining retractors, typically Caspar retractors, are introduced and an operating microscope is used for magnification. Following removal of scar tissue, flavectomy, and required bony decompression (i.e., arcotomy and/or partial medial facetectomy), the dural sac and nerve-root are carefully mobilized medially and the herniated disc evacuated. Removal of the disc herniation might involve entering the disc space or just removing a free sequestrated disc fragment (sequestrectomy).

### 2.6 Statistical analysis

Statistical analyses were performed with SPSS version 25.0 (IBM Corporation, Chicago, IL, USA). Statistical significance level was defined as p ≤0.05 on the basis of a two-sided hypothesis test with no adjustments made for multiple comparisons. Central tendencies are presented as means when normally distributed and as medians when skewed. We used the Chi square test for categorical variables. Baseline and one-year scores are compared with one-samples *t*-test for normally distributed data.

### 2.7 Missing data

Missing data for ODI, EQ-5D, NRS back and leg pain was handled with mixed linear models. This strategy is in line with studies showing that it is not necessary to handle missing data using multiple imputations before performing a mixed model analyses on longitudinal data (Twisk et al., 2013).

## 3 Results

### 3.1 Study population

In total, 276 patients were enrolled in the study. A total of 161 patients (58.3%) completed the one-year follow-up period. Baseline characteristics, surgical treatments, and comorbidities are summarized in Table 1. The mean patient age at baseline was 48.5 ​± ​13.3 years, and 38.4% were female. Non-responders were younger (44.2 vs 51.1) and had lower baseline ODI than responders (48.8 vs 53.2). Most patients (75%) only had one previous operation for lumbar disc herniation in the operated level.

**Table 1:** Baseline characteristics.

Variable	Value
Age at surgery (years), mean ​± ​SD	48.5±13.3
Female sex	106 (38.4%)
ASA > 2	34 (12.4%)
BMI, mean ​± ​SD	27±4.4
Obesity, BMI ≥ 30	48 (23.2%)
College education	95 (34.9%)
Daily tobacco smoking	100 (36.5%)
Mean preoperative ODI ​± ​SD	51.6±19.3
Mean preoperative EQ-5D	0.16±0.36
Preop. Leg pain NRS, mean ​± ​SD	7.2± 2.1
Preop. Back pain NRS, mean ​± ​SD	6.9±2.2
Spine level of surgery:	
L2-L3	5 (1.8%)
L3-L4	19 (6.9%)
L4-L5	130 (47.1%)
L5-S1	120 (43.5%)
Number of previous surgical procedures in the operated level (N ​= ​273)	
1	206 (74.6%)
2	49 (17.8%)
3	16 (5.8%)
4	2 (0.7)

### 3.2 Primary outcome

**Fig. 1:**
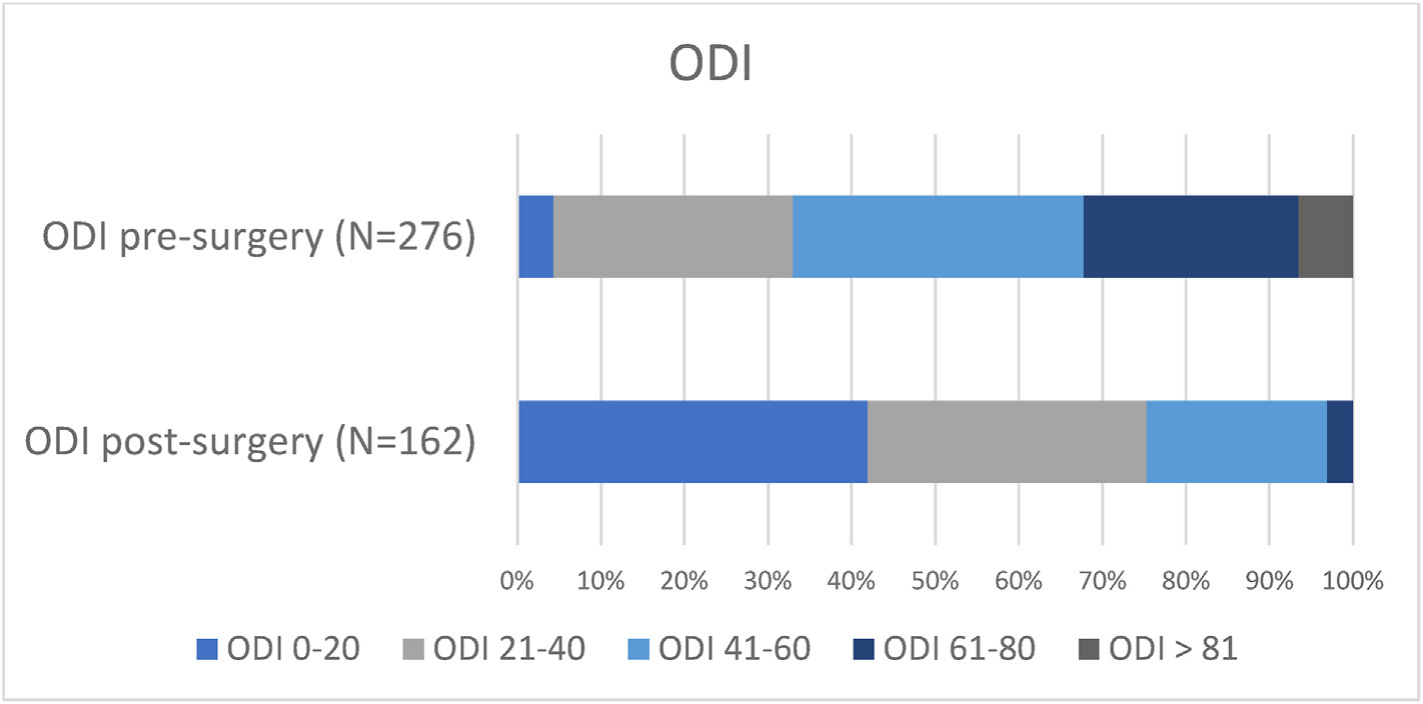
Case analysis of the group comparing ODI scores presurgery and twelve months after surgery. Data are presented in a stacked bar plot and table.

**Fig. 2:**
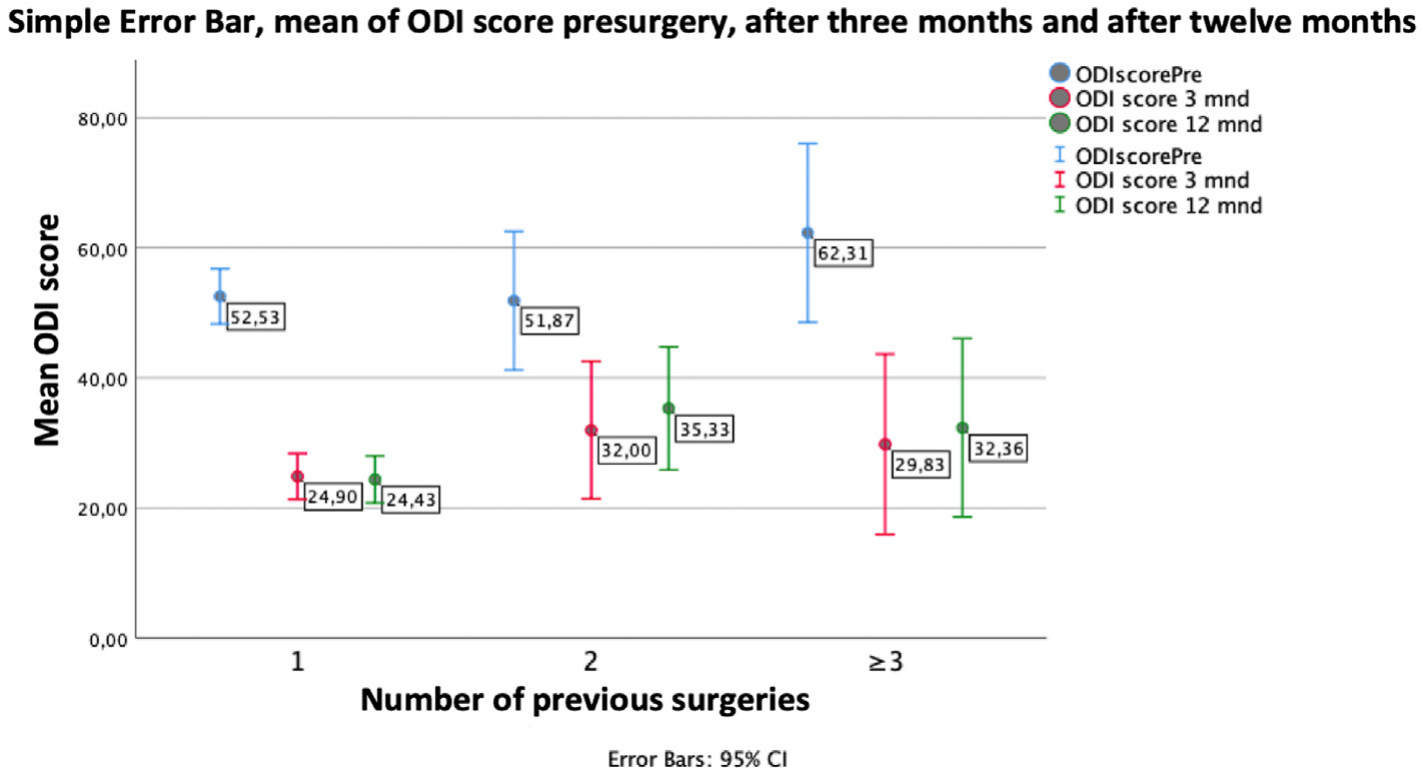
Oswestry disability index score at baseline, three months, and one year according to previous number of surgeries in the operated level. Error bars represent 95% confidence intervals.

Changes in ODI between baseline and one year after surgery are presented in Table 2. There was a significant improvement in the cohort between mean preoperative ODI and mean ODI at the one-year follow-up (27.1 points, 95% CI, 23.1 to 31.0; P <0.001). We performed a complete case analysis on the group that completed the one-year follow-up, presented in Fig. 1, Fig. 2. Among 161 patients with complete one-year follow-up, 105 patients (65.2%) experienced a clinically significant improvement (defined as an improvement of at least 13 ODI points). At one year 68 (42%) had an ODI score of 20 or less compared to 12 out of 275 patients (4.4%) at baseline. In total, 54 patients (33.5%) of the patients who completed the one-year follow-up experienced a change in ODI score of less than 33%. In addition, 25 patients (15.5%) had a raw ODI score of 48 or more or more after surgery.

**Table 2:** Patient reported outcome measures following lumbar microdiscectomy (complete case analyses).

Variable	Baseline	One year	Mean change	95% CI	P - Value
Oswestry disability index	53.6	26.5	27.1	23.1 to 31.0	<0.001
Euro-Qol 5D	0.13	0.60	−0.47	−0.54 to −0.40	<0.001
Leg pain NRS	7.2	3.8	3.4	2.8 to 3.9	<0.001
Back pain NRS	7.0	4.2	2.7	2.2 to 3.2	<0.001
**Mixed linear models:**					
Oswestry disability index	51.4	25.2	26.2	23.0 to 29.4	<0.001
Euro-Qol 5D	0.17	0.62	−0.44	−0.5 to −0.38	<0.001
Leg pain NRS	7.2	3.6	3.6	3.2 to 4.0	<0.001
Back pain NRS	6.9	4.1	2.8	2.4 to 3.2	<0.001

### 3.3 Secondary outcomes

Changes in EQ-5D, back pain NRS, and leg pain NRS at one year are presented in Table 2.

There was a significant difference between mean preoperative EQ-5D score and mean EQ-5D score at 1 year (0.47 points, 95% CI 0.40–0.54; P <0.001). An effect size of 1.3 was found for change in EQ-5D at one year, indicating a large clinical difference between the two time points.

The mean difference between the mean baseline value and one-year value in back pain NRS was 4.3 points (95% CI 2.2–3.2 ​P <0.001). Among patients with one-year follow-up, 94.3% experienced a clinically significant improvement (≥2 points).

The mean difference between the mean baseline value and one-year value in leg pain NRS was 3.8 points (95% CI 2.8–3.9, P <0.001), and 95.6% of the patients experienced a clinically significant improvement (≥2 points).

Mixed linear model analyses showed similar results for all patient-reported outcomes.

Complications are presented in Table 3. Out of the 276 patients included, nine (3.3%) experienced intraoperative complications, with unintentional durotomy as the most common complication (7 cases, 2.5% in total). Out of the 160 patients who completed the three-month follow up period, 15 (5.5%) experienced complications post-surgery following hospital discharge, with urinary tract infection as the most common complication (2.5%).

**Table 3:** Complications.

Perioperative complications no. (%)	9 (3.3%)
Unintentional durotomy	7 (2.5%)
Nerve injury	1 (0.4%)
Blood replacement	0
Cardiovascular complications	0
Anaphylactic reaction	0
Wrong level surgery	0
Respiratory complications	0
**Complications within 3 months no. (%) (N=160)**	**15 (5.5%)**
Wound infection	3 (1.1%)
Urinary tract infections	7 (2.5%)
Pneumonia	0
Pulmonary embolism	1 (0.4%)
Deep vein thrombosis	1 (0.4%)
Micturition problems	3 (1.1%)
**Reoperations (%)**	
Within 90 days:	37 (13.4%)

A total of 37 reoperations were performed within 90 days of the initial surgery, and 23 of these (62.2%) were due to residual LDH. One reoperation was due to hematoma.

## 4 Discussion

This study shows that microdiscectomy for recurrent LDH was associated with significant improvement across a wide range of patient reported outcome measures. Still, approximately one out of three patients did not achieve the desired improvement following surgery. The results from our study can be used to better inform patients about the likelihood of a successful surgical outcome and risks associated with repeat microdiscectomy.

In total, 65.2% experienced a clinically significant improvement defined as an improvement of at least 13 ODI points. Among the patients with complete one-year follow-up, 54 (33.5%) experienced a change in ODI score of less than 33%. In addition, 25 patients (15.5%) had a raw ODI score of 48 or more or more after surgery. These have been suggested as criteria with high accuracy for defining failure of surgery for lumbar disc herniation (Werner et al., 2017). Serious complications following microdiscectomy for recurrent LDH were fortunately rare, and the frequency of unintentional durotomies of 2.5% is substantially lower than what has been reported earlier (Guan et al., 2017).

Among the patients with complete follow-up, 42% experienced no or minimal disability at one year (i.e., an ODI score between zero and twenty). This is a lower proportion than previously reported in a study that excluded patients who had undergone previous spine surgery and found that 69.4% experienced no or minimal disability (Vangen-Lonne et al., 2020).

The mean improvement in ODI score of 27.1 points is less than what has been reported in both the SPORT trial (Koerner et al., 2015) and previous registry based observational studies (Lagerback et al., 2019; Elkan et al., 2016). These studies all excluded patients who had undergone previous lumbar spine surgery in the same level. This seems to suggest that previous surgical procedures have a negative impact on improvement. A study conducted in Sweden examining operations for recurrent LDH using data from the SWEspine register showed similar mean ODI score at baseline (51 points), and similar mean change in ODI (24 points) after a two-year follow-up period, but with a smaller sample size (Fritzell et al., 2015).

Studies utilizing the Japanese Orthopaedic Association score have reported a percentagewise improvement ranging between 52 and 65% (Fujiwara et al., 2003; Drazin et al., 2016; Guo et al., 2009). These studies all showed positive results in pain relief after surgery for recurrent LDH. As these studies used different outcome measures, had significantly smaller sample sizes, and included multiple surgical techniques, direct comparisons to our study are challenging (Drazin et al., 2016; Guo et al., 2009; El Shazly et al., 2013).

Previous studies have explored whether factors such as older age, obesity, duration of pain, and smoking influence outcomes following lumbar microdiscectomy (Madsbu et al., 2017, 2018a, 2018b; Vangen-Lonne et al., 2020; Nygaard et al., 2000). In patients undergoing microdiscectomy for LDH, promising results with decreased risk of reherniation and reoperation have been reported for the addition of a bone-anchored annular closure device in patients with large annular defects (Kursumovic and Rath, 2018; van den Brink et al., 2019). We found that the number previous surgeries clearly impacted functional outcomes. Information about the time interval from previous surgery to recurrent surgery was unavailable and may also impact patient reported outcome measures. Reasons for unsuccessful surgery with remaining disability in our study are probably multifaceted and additional factors such as patient expectations, comorbidity, lifestyle factors, demographic variables, coping strategies, correlation between image findings and symptoms, epidural fibrosis, number of previous surgeries, surgical strategy, and postoperative instability might influence results (Ebeling et al., 1989; Nerland et al., 2015b; Brinjikji et al., 2015). The two main surgical options for recurrent LDH are revision discectomy and instrumented fusion. There is no level I evidence demonstrating superiority of one approach over another (Hlubek and Mundis, 2017). A common understanding is that recurrent LDH is due to inherent instability and that this may be further increased by revision discectomy. Fusion is considered a reasonable option for recurrent LDH in the presence of instability and spinal deformity. In the absence of these indications, the choice of surgical strategy is more complicated. A US registry based study found similar clinical outcomes for repeat discectomy and fusion, but intraoperative blood loss, duration of surgery, length of hospitalization, and financial costs were clearly in favor of discectomy alone (Guan et al., 2017). A retrospective study assessing long-term outcomes following surgery for recurrent LDH also found similar outcomes for discectomy and fusion and the authors recommend discectomy alone as the initial surgical management (Fu et al., 1976). Our results add support to a treatment algorithm that favors discectomy as the first surgical intervention for recurrent LDH.

### 4.1 Strengths and limitations

Our study is strengthened by prospective data collection, high external validity, and widely applied and validated outcome measures (Fairbank et al., 1980; Grotle et al., 2003; Solberg et al., 2005; Ostelo et al., 1976; Twisk et al., 2013) Although this is a single center study, it is the largest to date with prospectively collected patient reported outcomes following surgery for recurrent LDH (Drazin et al., 2016). An obvious limitation is the lack of randomization as we did not have control groups that underwent non-surgical management or other surgical interventions than lumbar microdiscectomy. Another limitation is the lack of objective clinical outcomes (i.e. neurological and radiological outcome parameters). Our study is limited by a relatively high loss to follow-up (41.1%) at one year for the primary outcome measure, despite non-responders receiving reminders. Missing data in spine registries remain a concern and may introduce bias (van Hooff et al., 2015). However, a previous study examining a similar population with 22% loss to follow-up found no difference between responders and non-responders on long-term follow-up (Solberg et al., 2011). The use of mixed linear models in the management of missing data did not alter the results. There is also a possibility that some patients were operated again without consenting to further NORspine follow-up.

## 5 Conclusion

This study shows that microdiscectomy for recurrent LDH was associated with significant improvement across a wide range of patient reported outcome measures. Still, approximately one out of three patients did not achieve the desired improvement following surgery. The safety profile of lumbar microdiscectomy for recurrent LDH seems to be acceptable.
